# SEL1L deficiency impairs growth and differentiation of pancreatic epithelial cells

**DOI:** 10.1186/1471-213X-10-19

**Published:** 2010-02-19

**Authors:** Shuai Li, Adam B Francisco, Robert J Munroe, John C Schimenti, Qiaoming Long

**Affiliations:** 1 Department of Animal Science, College of Agriculture and Life Sciences, Cornell University, Ithaca, NY 14850, USA; 2 Department of Biomedical Sciences, College of Veterinary Medicine, Cornell University, Ithaca, NY 14850, USA

## Abstract

**Background:**

The vertebrate pancreas contains islet, acinar and ductal cells. These cells derive from a transient pool of multipotent pancreatic progenitors during embryonic development. Insight into the genetic determinants regulating pancreatic organogenesis will help the development of cell-based therapies for the treatment of diabetes mellitus. *Suppressor enhancer lin12/Notch 1 like (Sel1l*) encodes a cytoplasmic protein that is highly expressed in the developing mouse pancreas. However, the morphological and molecular events regulated by *Sel1l *remain elusive.

**Results:**

We have characterized the pancreatic phenotype of mice carrying a gene trap mutation in *Sel1l*. We show that *Sel1l *expression in the developing pancreas coincides with differentiation of the endocrine and exocrine lineages. Mice homozygous for the gene trap mutation die prenatally and display an impaired pancreatic epithelial morphology and cell differentiation. The pancreatic epithelial cells of *Sel1l *mutant embryos are confined to the progenitor cell state throughout the secondary transition. Pharmacological inhibition of Notch signaling partially rescues the pancreatic phenotype of *Sel1l *mutant embryos.

**Conclusions:**

Together, these data suggest that *Sel1l *is essential for the growth and differentiation of endoderm-derived pancreatic epithelial cells during mouse embryonic development.

## Background

The multiple cell types that make up the adult pancreas, including endocrine, exocrine and ductal cells, derive from a common pool of pancreatic progenitors. Pancreatic development in mice begins at embryonic day 9.5 (E9.5) with the formation of two epithelial buds on the dorsal and ventral side of the primitive gut endoderm [1]. Epithelial cells within the pancreatic buds proliferate rapidly and branch out during later embryonic days to form a complex tubular network comprised of undifferentiated multipotent progenitor cells [2,3]. Starting at E13.5, the expanded pancreatic epithelial cells undergo an asynchronized wave of differentiation to give rise to all the differentiated cell types of the adult pancreas, including acinar cells that produce hydrolytic digestive enzymes and islet cells that secrete endocrine hormones [4,5]. Pancreatic morphogenesis depends on a complex and yet incompletely characterized network of transcription factors. Significant efforts have been made in the past few years to understand the role of several important transcription factors, including *Pdx1 *[6,7], *Ptf1a *[8,9], *Sox9 *[10,11]9, *Ngn3 *[12,13], *NeuroD1 *[14,15], *Pax4 *[16], *Pax6 *[17], *Nkx2.2 *[18], *Nkx6.1 *[19], *Arx *[20], *Isl1 *[21] and *Insm1 *[22]. It is generally accepted that these transcription factors coordinate pancreatic morphogenesis by functioning in concert to restrict the developmental potentials of the pancreatic progenitors in a spatial and stage-specific manner [23].

Several previous studies have underscored the importance of Notch-mediated signaling in regulating pancreatic cell proliferation and cell fate decisions through control of *Ngn3 *gene expression. During pancreatic development, *Ngn3 *is transiently expressed in a subset of the pancreatic epithelial cells. NGN3 deficiency completely abolishes formation of all the endocrine cell subtypes, suggesting Ngn3 functions as a master switch for the endocrine lineage in the pancreas. Mutations in genes encoding Notch signaling pathway components, such as DLL1 (ligand), RBP-Jk (the intracellular mediator), or HES-1 (the effector) causes expansion of Ngn3 expression in pancreatic cells and, as a result, accelerated differentiation of endocrine cells at the expense of acinar and ductal cells [13,24,25]. Conversely, over or persistent expression of the Notch intracellular domain (NICD), a constitutively active form of Notch receptors, or the Notch effector gene *Hes1 *results in diminished expression of *Ngn3 *and attenuated differentiation of endocrine cells [26-28]. These studies suggest that during pancreatic development Notch signaling controls the endocrine and exocrine cell fate decisions of pancreatic epithelial cells by directly regulating *Ngn3 *expression. Recent studies have also indicated the importance of Notch signaling in control of exocrine cell differentiation. Ectopic expression of activated NOTCH-1 prevents or significantly delays differentiation of acinar cells [26,27].

While the role of Notch signaling in control of pancreatic cell proliferation and cell fate decisions is clearly recognized, the molecular mechanisms necessary for proper control of Notch signaling during vertebrate pancreatic development are poorly understood. Genetic and biochemical studies in invertebrates suggest that regulation of Notch signaling occurs at various levels and through multiple mechanisms [29-31]. These include stochastic and/or developmental expression of the Notch receptors and their ligands [32-34], selective receptor-ligand interactions [35,36], intracellular protein trafficking [37] and stability of NICD [38]. *Suppressor enhancer lin12 1 like (Sel1l*) encodes a cytoplasmic protein that is highly conserved throughout the vertebrate kingdom [39]. RNA *in situ *hybridization and immunohistological analysis revealed that *Sel1l *is highly expressed in both the embryonic and adult pancreas [40-42]. The human *Sel1l *gene is located in a chromosome region that is in close proximity to a type 1 diabetes high risk locus, IDDM-11 (insulin-dependent diabetes mellitus locus 11), prompting the speculation that mutations in *Sel1l *may be associated with the pathogenesis of type 1 diabetes [43]. *Sel-1*, the *C. elegans *ortholog of *Sel1l*, was first identified in a genetic screen for mutations that suppress lin-12/Notch activity [44]. Subsequent biochemical studies demonstrated that *Sel-1 *negatively regulates lin-12/Notch activity by controlling lin-12/Notch turn-over [45,46]. Based on these findings, it has been suggested that *Sel1l *may also function as a negative regulator for Notch signaling [47]. Recent biochemical and molecular studies *in vitro *revealed that *Sel1l *is also required for maintaining homeostasis of the endoplasmic reticulum (ER). SEL1L nucleates an ER membrane protein complex that is required for dislocation of unfolded or misfolded proteins from the ER lumen into the cytosol for degradation [48,49].

In an attempt to decipher the developmental and physiological roles of *Sel1l*, we have generated and characterized mice carrying a gene trap mutation in the *Sel1l *gene. We report here that *Sel1l *expression during pancreas development coincides with differentiation of pancreatic epithelial cells into both the endocrine and exocrine lineages. Homozygous *Sel1l *mutant embryos exhibit an impaired pancreatic epithelial growth and branching morphology. Pharmacological inhibition of Notch signaling rescues the pancreatic phenotype of *Sel1l*-deficient embryos. These data are consistent with the notion that *Sel1l *functions as a negative regulator for Notch signaling during pancreatic organogenesis [47].

## Results

### *Sel1l *expression during mouse pancreatic development

**Figure 1:**
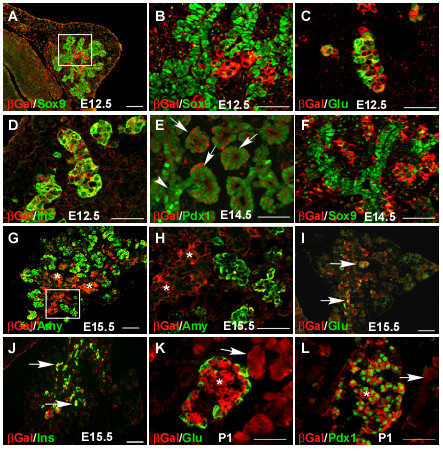
**Spatiotemporal expression of *Sel1l *during development of the mouse pancreas**. Pancreatic sections from timed *Sel1l*^+/*βgeo *^embryos were co-immunostained with antibodies against βGal (red) and various pancreatic markers (green) as indicated on the left side of each panel. (**B **and **H**) Magnified views of the boxed area in **A **and **G**, respectively. (**A-D**) βGal expression at E12.5. βGal expression begins in a small number of cells within the core pancreatic epithelium at E12.5 (**A**). At this stage, there is no co-expression of βGal and SOX9 (**B**); however, βGal is co-expressed with either glucagon (**C**) or insulin (**D**). (**E-F**) βGal expression at E14.5. βGal is differentially expressed in the PDX1^+ ^cells (**E**). While high βGal signal was detected in the epithelial branches (arrows), no or very low Gal signal was detected in the core "duct-like" epithelium (arrowhead). These core epithelial cells correspond to the subset of PDX^+ ^cells that express SOX9 (**F**). (**G-J**) βGal expression at E15.5. βGal expression increases markedly in the core pancreatic epithelium (**G **and **H, **asterisks). βGal co-localizes with either glucagon (**I, **arrows) or insulin (**J, **arrows). (**K-L**) βGal expression at postnatal day 1. βGal is expressed in both islet and acinar tissues (**K, **asterisk and arrow, respectively). βGal co-localizes with PDX1 in the islet tissue (**L**). Scale bars: (**A**, **G**, **I **and **J**) 50 μm; (**B-F**, **K-L**) 100 μm.

We generated mice carrying a gene-trap insertion in the *Sel1l *gene. The gene trap cassette, located in intron 14, contains a β-galactosidase-neomycin (βgeo) fusion reporter gene. To determine the spatiotemporal expression pattern of *Sel1l *in the developing mouse pancreas, we performed immunohistological analysis of pancreatic sections from *Sel1l*^+/*βgeo *^embryos using antibodies against β-galactosidase (βgal) and several pancreatic proteins, including SOX9 and PDX1 (progenitor markers), insulin and glucagon (endocrine lineage markers) and amylase (exocrine lineage marker). βgal expression was first detected at E12.5 in a small number of cells located within the core of SOX9-expressing pancreatic epithelium (Fig. 1A-B). The βgal^+ ^cells at E12.5 express either glucagon (Fig. 1C) or insulin (Fig. 1D), indicating that they were early endocrine cells. βgal expression expands significantly between E12.5 and E14.5. At E14.5, differential βgal expression was detected in the PDX1^+ ^cells throughout the pancreatic epithelium (Fig. 1E). Epithelial cells with markedly lower βgal expression correspond to a subset of PDX1^+ ^cells that express SOX9 (Fig. 1F). At E15.5, high βgal expression was detected in the core of the differentiating pancreas (Fig. 1G-H, asterisks). These βgal^+ ^cells were positive either for glucagon or insulin (arrows in Fig. 1I and 1J, respectively). At birth, βgal expression was detected in both islet and acinar cells (Fig. 1K-L, asterisk and arrow, respectively). Taken together, these expression data indicate that *Sel1l *is selectively expressed in the differentiating or differentiated pancreatic epithelial cells.

### SEL1L deficiency results in defective pancreatic epithelial growth and branching morphogenesis

**Figure 2:**
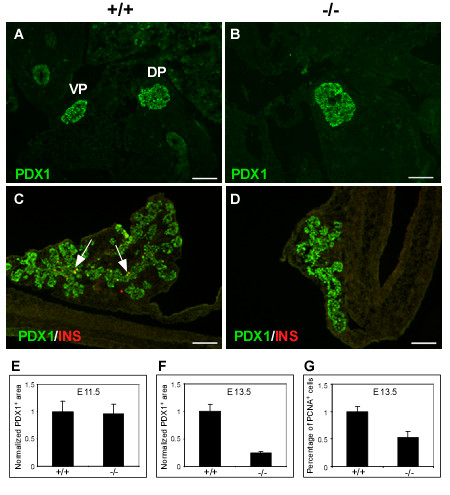
**Impaired pancreatic epithelial growth and branching morphogenesis in *Sel1l***^*β**geo***/*β**geo ***^**embryos**. (**A-D**) Immunohistological analysis of the developing pancreas of wild-type and *Sel1l*^*βgeo*/*βgeo *^embryos at E11.5 and E13.5; the genotypes of the pancreatic sections are indicated as +/+ and -/-, respectively. The following antibodies were used: Pdx1 (**A-D**, green) and insulin (**C-D**, red). (**A-B**) The dorsal and ventral pancreatic buds in *Sel1l*^*βgeo*/*βgeo *^embryos are fused together. (**C-D**) The dorsal pancreatic bud of *Sel1l*^*βgeo*/*βgeo *^embryos exhibits a markedly reduced epithelial size and an impaired branching morphology. (**E-F**) Statistical analyses of estimated pancreatic epithelial sizes of wild-type and *Sel1l*^*βgeo*/*βgeo *^embryos at E11.5 (**E**) and E13.5 (**F**). Data were from three sets of wild-type and *Sel1l*^*βgeo*/*βgeo *^embryos. No significant difference in the epithelial sizes of wild-type and *Sel1l*-deficient pancreas at E11.5 was detected (**E**). At E13.5, the pancreatic epithelium of *Sel1l*-deficient embryos was significantly smaller (**F**). Scale bar: 100 μm.

The gene trap in *Sel1l *contains a strong splicing acceptor signal that efficiently blocks splicing between exon 14 and 15, resulting in a truncated Sel1l transcript. Homozygous gene trap mice *(Sel1l*^*βge*/*βgeo*^) exhibit systemic endoplasmic reticulum stress and die before E13.5 to E15.5 (Additional file 1). To determine if *Sel1l *is required for pancreatic epithelial growth, we performed morphometrical analysis of the developing pancreas of viable *Sel1l*^*βgeo*/*βgeo *^embryos. At E11.5, the dorsal and ventral pancreatic buds in *Sel1l*^*βgeo*/*βgeo *^embryos appeared to be closely linked (Fig. 2B), as compared to those in wild-type embryos (Fig. 2A). There was no significant difference in the pancreatic epithelial size between wild-type and *Sel1l*^*βgeo*/*βgeo *^embryos (Fig. 2E). At E13.5, the dorsal pancreatic bud of *Sel1l*^*βgeo*/*βgeo *^embryos (Fig. 2C) was clearly smaller than that of wild-type embryos (Fig. 2F). In addition, while wild-type pancreatic epithelium displayed a well-branched structure, mutant pancreatic epithelium appeared to be a simple tube of epithelial cells. TUNEL assays revealed no significant increase of apoptosis in the developing pancreas of *Sel1l *mutant embryos (data not shown). Immunostaining using anti-PCNA (a cell proliferation indicator) antibody indicated that the pancreatic epithelium of *Sel1l *mutant embryos had a significantly lower rate of cell proliferation than wild-type pancreatic epithelium (Fig. 2G). Together, these results indicate that *Sel1l*, while dispensable for pancreatic epithelial induction, is essential for the subsequent growth and branching morphogenesis of the pancreatic epithelial cells.

### SEL1L deficiency inhibits differentiation of acinar cells and significantly attenuates differentiation of endocrine cells

**Figure 3:**
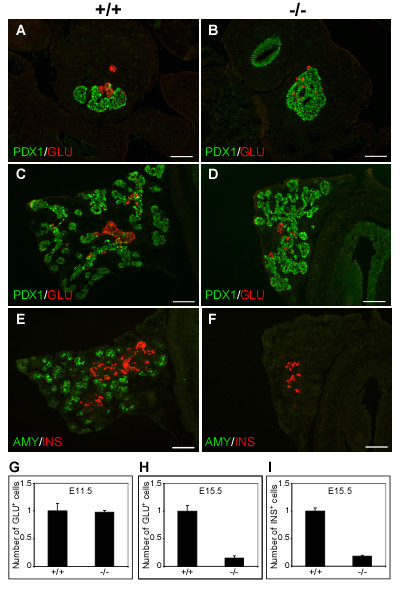
**Inhibited endocrine and exocrine cell differentiation in *Sel1l*^*βgeo*/*βgeo *^embryos**. (**A-F**) Immunohistological analysis of the developing pancreas of *Sel1l*^+/+ ^and *Sel1l*^*βgeo*/*βgeo *^embryos at E11.5 (**A-B**) and E15.5 (**C-F**); the genotypes of the pancreatic sections are indicated as "+/+" and "-/-", respectively. The following antibodies were used: Pdx1 (**A-D**, green), glucagon (**A-D**, red), insulin (**E-F**, red) and amylase (**E-F**, green). (**A-B**) At E11.5, Glu^+ ^cells were detected in the pancreatic epithelium of wild-type and *Sel1l*^*βgeo*/*βgeo *^embryos in equal numbers. (**C-D**) At E15.5, the number of glucagon^+ ^cells was reduced in *Sel1l*^*βgeo*/*βgeo *^embryos as compared to wild-type control. (**E-F**) No amylase^+ ^cells were detected and the number of insulin^+ ^cells was significantly reduced in *Sel1l*^*βgeo*/*βgeo *^embryos. (**G-I**) Statistical analyses of the numbers of glucagon^+ ^and insulin^+ ^cells in wild-type and *Sel1l*^*βgeo*/*βgeo *^embryos. Data were from three wild-type and three *Sel1l*^*βgeo*/*βgeo *^embryos. At E11.5, no significant difference in the number of glucagon^+ ^cells in wild-type and *Sel1l*^*βgeo*/*βgeo *^embryos was detected (**G**). The number of glucagon^+ ^and insulin^+ ^cells was markedly lower in *Sel1l*^*βgeo*/*βgeo *^embryos than in wild-type control embryos (**H **and **I**, respectively). Scale bar: 100 μm.

We next investigated the role of *Sel1l *in pancreatic epithelial cell differentiation. Since the first wave of cell differentiation occurs before E12.5 and the cells generated are mostly α-cells, we analyzed glucagon expression in E11.5 *Sel1l*^*βgeo*/*βgeo *^embryos. As shown in Fig. 3A-B and 3G, comparable numbers of glucagon^+ ^cells were observed in wild-type and *Sel1l*^*βgeo*/*βgeo *^embryos. *Sel1l *is thus unlikely to be required for the generation of early endocrine cells during the first transition (E9.5 to E12.5).

**Figure 4:**
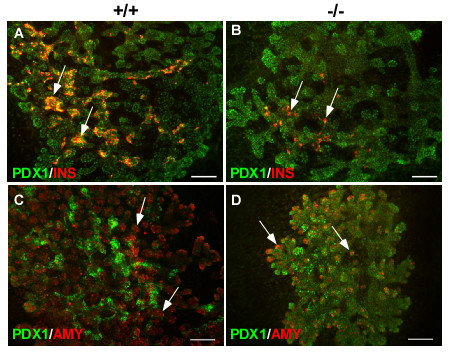
***Sel1l*-deficient pancreatic epithelium exhibits impaired growth, branching morphology and differentiation *ex vivo***. Dorsal pancreatic buds of wild-type and *Sel1l*^*βgeo*/*βgeo *^embryos were isolated at E11.5 and cultured as described in Materials and Methods. The genotypes of the pancreatic explants are indicated as "+/+" and "-/-", respectively. Cultured pancreatic explants were immunostained using the indicated antibodies. *Sel1l*-deficient pancreatic epithelium grows poorly and displays impaired branching morphology (**B **and **D**), as compared to wild-type control explants (**A **and **C**). The number of insulin+ cells in mutant pancreatic epithelia were markedly reduced (**B, **arrows), as compared to the wild-type controls (**A, **arrows). Amylase expression was detected in *Sel1l*-deficient pancreatic epithelium (**D**, arrows), but the expression is significantly lower than that in wild-type epithelium (**C**, arrows). Scale bar: 100 μm.

While the majority of *Sel1l*^*βgeo*/*βgeo *^embryos (95%) die before the initiation of the major wave of cell differentiation in the developing pancreas (E13.5), about 5% of these embryos are viable at E15.5. To determine if *Sel1l *is required for differentiation of pancreatic epithelial cells during the secondary transition (E13.5 to E15.5), we analyzed the expression of three pancreatic lineage markers: insulin (β-cells), glucagon (α-cells) and amylase (acinar cells) in these embryos. Significant numbers of glucagon^+ ^and insulin^+ ^cells were detected in wild-type embryos at E15.5 (Fig. 3C and 3E). In contrast, the numbers of glucagon^+ ^and insulin^+ ^cells were significantly reduced in *Sel1l*^*βgeo*/*βgeo *^embryos (Fig. 3D and 3F, 3H and 3I). Abundant amylase^+ ^cells are present in wild-type embryos at E15.5 (Fig. 4E). In contrast, no amylase^+ ^cells are detectable in *Sel1l*^*βgeo*/*βgeo *^embryos (Fig. 4F). Disruption of *Sel1l *function thus results in an impaired differentiation of both acinar and islet cells.

### SEL1L-deficient pancreatic epithelium exhibits inhibited growth and differentiation *ex vivo*

To ensure that the growth and differentiation defects observed in *Sel1l*^*βgeo*/*βgeo *^embryos were not due to the effect of global embryonic growth retardation of these embryos, we studied the pancreatic phenotype of *Sel1l*^*βgeo*/*βgeo *^embryos using an organ culture system. The dorsal pancreatic bud of wild-type and *Sel1l*^*βgeo*/*βgeo *^embryos were isolated at E11.5 and cultured for 8 days. The cultured pancreatic explants were then analyzed by immunofluorescence using antibodies against PDX1 and insulin. In general, *Sel1l*-deficient pancreatic epithelium grows poorly (Suppl. Fig. 2B and 2D) with about half of the cultured mutant epithelia failing to form branched epithelial structures. This is in sharp contrast to the growth behavior of wild-type pancreatic epithelia, which form a branched epithelial structure (Additional file 2). The number of insulin^+ ^cells was significantly decreased in *Sel1l*-deficient pancreatic explants (Fig. 4B), as compared to that in wild-type control explants (Fig. 4A). Amylase^+ ^cells were detected in cultured *Sel1l*-deficient explants (Fig. 4D), although the number of amylase^+ ^cells was significantly lower compared to that in wild-type control explants (Fig. 4C). Thus, the pancreatic epithelium of *Sel1l*^*βgeo*/*βgeo *^embryos displays impaired growth, branching morphogenesis and lineage differentiation *ex vivo*. These findings confirm that the pancreatic defects observed in *Sel1l*-deficient embryos at E15.5 are not due to the global growth retardation.

### Pancreatic progenitor cells in SEL1L-deficient embryos at E15.5 are confined to a pluripotent progenitor state

**Figure 5:**
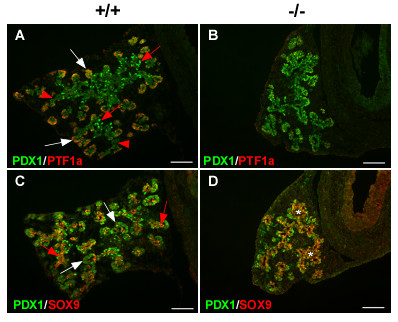
***Sel1l*-deficient pancreatic epithelial cells fail to commit to lineage precursors during the secondary transition**. (**A-D**) Immunohistological analysis of the developing dorsal pancreas of wild-type and *Sel1l*^*βgeo*/*βgeo *^embryos at E15, using the indicated antibodies. The genotypes of the pancreatic sections are indicated as "+/+" and "-/-", respectively. (**A-B**) PTF1a is expressed in the periphery of wild-type pancreatic epithelium marking the commitment of pancreatic epithelial cells into the exocrine lineage (**A**, white arrows); PTF1a expression is absent in the mutant pancreas (**B**). Up-regulation of PDX1 expression in a subset of epithelial cells in the core of wild-type pancreatic epithelium marks the commitment of pancreatic epithelial cells into the endocrine lineage (**A**, red arrows); whereas PDX1 is uniformly expressed in mutant pancreatic epithelial cells (**B**). (**C-D**) SOX9 is expressed in a small population of epithelial cells in wild-type pancreatic epithelium (**C**, red arrows); in contrast, SOX9 mostly co-localizes with PDX1 in mutant pancreatic epithelium (**D**, asterisks). Scale bar: 100 μm.

To gain insight into the molecular basis underlying the pancreatic defects in *Sel1l*^*βgeo*/*βgeo *^embryos, we first analyzed the expression of several transcription factors important for pancreatic epithelial growth and lineage formation. Immunostaining was carried out to assess the expression of *Sox9, Pdx1 *and *Ptf1a *in E15.5 *Sel1l*^*βgeo/βgeo *^embryos. PDX1 expression was uniformly detected in the pancreatic epithelial cells of *Sel1l*^*βgeo/βgeo *^embryos (Fig. 5B). This is in sharp contrast to wild-type embryos where PDX1 expression was differentially expressed in the pancreatic epithelial cells, with high expression in a subset of the core epithelial cells and low expression in the peripheral epithelial cells (Fig. 5A, white arrow and red arrowheads, respectively). No PTF1a expression was detected in the pancreatic epithelium of *Sel1l*^*βgeo/βgeo *^embryos (Fig. 5B), whereas clear co-expression of PTF1a and PDX1 was detected in the proacini in the periphery of the pancreatic epithelial buds (Fig. 5A, white arrows). SOX9 was detected in approximately 20% of the PDX1^+ ^cells in wild-type embryos (Fig. 5C, red arrows). In contrast, SOX9 expression was observed in more than 70% of the PDX1^+ ^cells in *Sel1l*^*βgeo/βgeo *^embryos (Fig. 5D, asterisk). These findings are consistent with the notion that the pancreatic epithelial cells in *Sel1l*^*βgeo/βgeo *^embryos at E15.5 are restricted the pancreatic progenitor state.

### Pharmacological inhibition of Notch signaling rescues endocrine lineage formation in *Sel1l*^*βgeo/βgeo *^embryos

**Figure 6:**
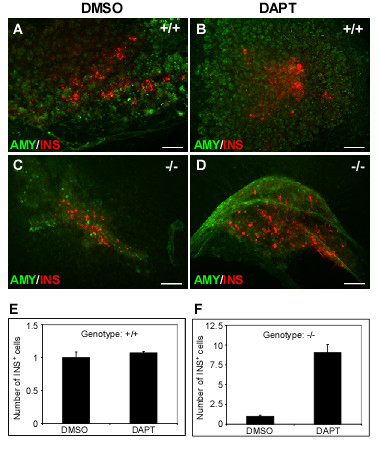
**DAPT treatment rescues the pancreatic phenotype of *Sel1l*^*βgeo*/*βgeo *^embryos**. The dorsal pancreatic bud of wild-type (+/+) and *Sel1l *mutant (-/-) embryos was isolated at E11.5 and cultured for 8 days in the presence of 0.1% DMSO (control) and 10 μM of DAPT. (**A-D**) Immunohistological analysis of DAPT-treated and non-treated pancreatic buds with the indicated antibodies. (**E-F**) Quantification of insulin^+ ^cells in DMSO and DAPT-treated pancreatic explants. DAPT did not significantly change the number of insulin^+ ^cells in wild-type pancreatic buds (**A-B **and **E**), however, it induced a marked increase of insulin^+ ^cells in *Sel1l *mutant pancreatic buds (**C-D **and **F**). Scale bar: 100 μm.

*Sel1l *was thought to be a negative regulator for Notch signaling [47]. Indeed, the pancreatic phenotype in *Sel1l*^*βgeo/βgeo *^embryos shows remarkable similarity to that of mouse or zebrafish embryos over-expressing Notch intracellular domain, the constitutively active form of Notch receptors [26,28,50]. We speculated that the impaired pancreatic epithelial growth and differentiation in Sel1l-deficient embryos may be due to an increased Notch signaling activity. To test this possibility, we used the γ-secretase inhibitor, DAPT (Difluorophenacetyl-al-alanyl-S-phenylglycine-t-butyl ester), to suppress Notch signaling in pancreatic explants culture. The dorsal pancreatic bud of wild-type and *Sel1l*^*βgeo/βgeo *^embryos was isolated at E11.5 and cultured for 8 days in the absence or presence of DAPT. At a concentration of 10 μM, DAPT did not exhibit detectable effects on the growth, branching morphology and endocrine cell differentiation of wild-type pancreatic epithelium (Fig. 6A-B and 6E). At the same concentration, however, DAPT caused a significant expansion of the SEL1L-deficient pancreatic epithelium (Fig. 6C-D). Importantly, DAPT treatment resulted in a marked increase in the number of insulin^+ ^cells (Fig. 6D and 6F). These observations indicate that pharmacological inhibition of Notch signaling rescues, at least partially, the pancreatic epithelial growth and endocrine differentiation defects of *Sel1l*^*βgeo/βgeo *^embryos.

## Discussion

The genetic determinants underlying vertebrate pancreatic development are not completely understood. In the present study, we report that *Sel1l *expression in the developing mouse pancreas coincides with differentiation of the endocrine and exocrine lineages during the second transition. Embryos homozygous for a gene trap insertion in *Sel1l *display impaired pancreatic epithelial growth and differentiation. Pharmacological inhibition of Notch signaling partially rescues the pancreatic phenotype of SEL1L-deficient embryos. Together, these data provide evidence that *Sel1l *is required for growth and differentiation of the mammalian pancreatic epithelial cells. The underlying mechanism may be that *Sel1l *functions as a negative regulator for the Notch signaling pathway by facilitating degradation of the Notch intracellular domain.

Donoviel *et. al*. previously reported that the mouse *Sel1l *gene is highly transcribed in the acini of the developing pancreas at E14.5 and E17.5 [41]. We show, by immunohistological analysis of the β-galactosidase reporter in *Sel1l*, that *Sel1l *is also highly expressed in early glucagon^+ ^and insulin^+ ^cells (Fig. 1C and 1D, respectively) generated during the first transition period of pancreatic development [5,51,52]. Consistent with the *Sel1l *mRNA expression pattern reported by Donoviel *et. al*., we reveal that at E14.5 *Sel1l *is broadly expressed in the PDX1^+ ^pancreatic epithelia (Fig. 1E). Interestingly, significantly lower *Sel1l *expression is observed in the undifferentiated pancreatic epithelial cells (Fig. 1F) that express SOX9 [10,11]. Our findings therefore indicate that *Sel1l *expression is enhanced in the committed pancreatic epithelial cells or differentiated endocrine and exocrine cells.

The selective expression of *Sel1l *in differentiating or differentiated pancreatic epithelial cells is highly suggestive of a role for *Sel1l *in promoting lineage differentiation during the secondary transition. To define the role of *Sel1l *in pancreatic development, we characterized the pancreatic phenotype of embryos homozygous for a gene trap insertion in *Sel1l*. We show that wild-type and *Sel1l*-deficient embryos have a comparable pancreatic epithelial size at E11.5 (Fig. 2A-B and 2E), suggesting that *Sel1l *function is dispensable for the induction and early growth of pancreatic epithelial cells. At E13.5, however, *Sel1l*-deficient pancreatic epithelia are significantly smaller and display an impaired branching morphology (Fig. 2C-D and 2F). Importantly, *Sel1l*-deficient pancreatic epithelia at E15.5 completely lack amylase^+ ^cells (Fig. 3F) and have significantly reduced numbers of glucagon^+ ^and insulin^+ ^cells (Fig. 3D and 3F, respectively). Also, by analyzing the phenotype of cultured *Sel1l*-deficient dorsal pancreatic epithelia, we confirmed that the pancreatic defects in *Sel1l*-deficient embryos are not due to the effect of global growth retardation of the *Sel1l*-deficient embryos (Fig. 4A-D). Together, these data strongly suggest that *Sel1l *plays an important role in mouse pancreatic organogenesis by facilitating differentiation of the endocrine and exocrine lineages of the pancreas.

The *C. elegans *ortholog of the mammalian *Sel1l *gene, *Sel-1*, was originally identified by genetic screening as a negative regulator for lin-12/Notch signaling [44,53]. Biochemical studies have shown that *Sel-1 *regulates lin-12/Notch receptor turnover [45,46]. Whether or not Sel1l has similar functions in the mammalian system remains unclear [54]. In this study, we provide two lines of evidence that suggest *Sel1l *also functions as a negative regulator for Notch signaling, at least in the developing mouse pancreas. First, the observed pancreatic phenotype in *Sel1l *mutant embryos show remarkable similarity to the pancreatic phenotype of transgenic vertebrate embryos over-expressing the Notch intracellular domain, the constitutively active form of Notch [26,28,50]. Second, we show that treatment of cultured *Sel1l*-deficient pancreatic epithelium with DAPT, a potent Notch signaling inhibitor, partially rescues the growth and differentiation defects of *Sel1l*-deficient pancreatic epithelium (Fig. 6D and 6F).

How does SEL1L negatively control Notch signaling in the developing mammalian pancreas is the focus of our current investigation. Genetic and biochemical evidences from other organisms such as *Drsophila *and *C. elegans *suggest that Notch signaling is regulated at multiple levels and through multiple mechanisms, including stochastical gene expression [34], selective ligand-receptor interactions [35], intracellular trafficking [37], and stability of NICD, the active form of Notch receptors [30]. Given the previous finding that mammalian NICD are ubiquitinated and degraded through both the proteasomal and lysosomal systems [55,56], it is conceivable that Sel1l may be directly or indirectly involved in control of NICD degradation. In this regard, it is noteworthy to mention that recent biochemical data from our laboratory indicate that NICD has significantly higher protein stability in *Sel1l*-deficient MEF cells (data not shown). Further proof of this hypothesis will require a detailed biochemical analysis of SEL1L and Notch interactions.

Biochemical evidences from other laboratories have also underscored the importance of *Sel1l *in maintaining ER homeostasis. SEL1L interacts with the E3 ubiquitin ligase HRD1 to facilitate the dislocation of unfolded or misfolded proteins from the ER lumen into the cytosol for degradation [48,49,57-60]. Consistent with this, we have recently shown that homozygous *Sel1l *mutant mice develop systemic ER stress. The impact of ER stress on pancreatic epithelial proliferation and differentiation remains undefined from the current study. However, several hypothetical roles of ER stress in pancreatic organ development and growth can be proposed. First, ER stress affects global gene expression. Upon ER stress, translation is generally down to reduce production of ER client proteins. Concomitantly, transcription of genes encoding ER chaperones and ER-associated degradation (ERAD) machinery is increased. Second, ER stress blocks or reduces protein secretion by mammalian cells. This will directly affect cell signaling and cell functions. Finally, prolonged ER stress activates the signaling pathways leading to apoptosis.

## Conclusions

We report that during mouse pancreatic development, *Sel1l *is preferentially expressed in the differentiating or differentiated pancreatic epithelial cells. Disruption of *Sel1l *function results in impaired endocrine lineage formation and delayed exocrine lineage differentiation. Pharmacological suppression of Notch signaling in cultured pancreatic explants partially rescues the endocrine cell differentiation defect. Our data suggest that *Sel1l *may regulate pancreatic epithelial growth and differentiation by suppressing Notch-mediated signaling.

## Methods

### *Sel1l *gene trap mice

The chimeric founder mouse used to generate S*el1l *gene trap mice was generated by microinjection of a commercially available mouse embryonic stem (ES) cell clone, CA0017, into C57/BL6 blastocysts. This ES cell clone contains a gene trap insertion in the 14^th ^intron of the *Sel1l *gene. All mice or mouse embryos were genotyped by PCR analysis of tail or toe genomic DNA using the following PCR primers:

F-Sel1l: 5'-TGGGACAGAGCGGGCTTGGAAT-3';

R-Sel1l: 5'-CACCAGGAGTCAAAGGCATCACTG-3';

R-βGeo: 5'ATTCAGGCTGCGCAACTGTTGGG-3'.

All animal experiments were performed in accordance with the Cornell Animal Care and Use Guidelines.

### Histology and immunohistochemistry

Embryos or pancreatic rudiments were fixed in 4% paraformaldehyde (PFA) in PBS at 4°C for 12 hours or longer. PFA-fixed specimens were equilibrated in 30% sucrose in PBS at 4°C and embedded in O.C.T. Sections were cut at 10 μm and immunostained essentially as described [61,62]. Briefly, tissue sections were permeabilized with 0.2% Triton X-100 in PBS for 20 min, washed 3 times in 0.1% Triton X-100 in PBS and 3 times in PBS. The permeabilized sections were pre-incubated with 5% normal donkey serum and 1% BSA in PBS at room temperature for at least 1 hour, followed by incubation in the same solution with primary antibodies at 4°C overnight. The antibody-bound sections were then washed three times in 0.1% Triton X-100 in PBS, 3 times in PBS and then incubated with secondary antibodies for 1-3 hrs at room temperature. Primary antibodies were diluted in 1% BSA in PBS as follows: rabbit anti-b-Galactosidase (Immunology Consultants Laboratory), 1:500; guinea pig anti-Pdx-1 (Abcam), 1:1000; goat anti-Pdx1 (Abcam), 1:1000; guinea pig anti-insulin (Linco), 1:1000; rabbit anti-glucagon (Covance), 1:500; goat anti-glucagon (AbD Serotec), 1:200; rabbit anti-Sox9 (gift from Dr. Michael Wegner), 1:1000; rabbit anti-Ptf1a (gift from Dr. Helena Edlund), 1:1000); and rabbit anti-amylase (Sigma), 1:500. Secondary antibodies were diluted in 1% BSA in PBS as follows: donkey anti-rabbit IgG conjugated to Cy3 (Jackson Immuno Research), 1:1000; donkey anti-guinea pig IgG conjugated to Cy2 (Jackson Immuno Research), 1:500. Fluorescence images were acquired using an Axiovert 40 microscope (Zeiss) equipped with an AxioCam camera.

### Pancreatic morphometry and cell counting

Quantitative morphometry of the developing pancreas and cell counting were performed essentially as described [11]. Briefly, pancreata of age-matched wild-type and *Sel1l*^*βgeo/βgeo *^embryos were sectioned through and every fifth section was immunostained with anti-PDX1 or co-immunostained with anti-PDX1 and anti-Glucagon. Quantification of pancreatic epithelium area (PDX1^+ ^area) was performed using AxioVision Imaging analysis software (version 4.6.3). The data were presented as averages ± SEM (μm^2^) of three independent pancreata. Statistical analysis was performed using the Student's two-sample *t *test and significance is regarded as p ≤ 0.05.

### Pancreatic organ culture and DAPT treatment

*Sel1l*^+/*βgeo *^mice were intercrossed to generate embryos of defined genotypes: wild-type (*Sel1l*^+/+^), heterozygous (*Sel1l*^+/*βgeo*^) and homozygous (*Sel1l*^*βgeo/βgeo*^). The day of the vaginal plug was taken as embryonic (E) day 0.5. Pancreatic bud dissection and culture were performed essentially as described [63]. Briefly, E11.5 embryos were decapitated and the dorsal pancreatic rudiment together with surrounding mesenchymal tissue was removed in PBS. The dissected pancreatic buds were rinsed once in culture medium, transferred to a fibronectin-coated 8-well LabTek chamber slide with the epithelial side touching the bottom of the slide and cultured for 72 hours in basal medium with Earle's salts (Gibco 21010-046), 1 × glutamine, 10% FBS and antibiotics. The cultured pancreatic rudiments were fixed individually for 30 minutes in 4% paraformaldehyde in PBS for immunohistological analysis. DAPT treatment of cultured pancreatic explants was performed essentially as described [64]. DAPT stock solution was prepared at a concentration of 10 mM in DMSO and was aliquoted into single-usage aliquots. For Notch inhibition experiments, pancreatic buds were cultured in complete medium containing 10 μM of DAPT. Control pancreatic buds were cultured in complete medium containing 0.1% DMSO. The DMSO or DAPT-treated pancreatic buds were briefly fixed and analyzed by immunostaining.

## Authors' contributions

QL designed the study and prepared the manuscript. RM and JS generated the gene trap mice. SL performed the immunohistochemistry, pancreatic bud culture and DAPT rescue experiments. AF maintained the gene trap mice and performed genotyping analysis. All authors reviewed and approved the manuscript.

## Supplementary Material

Additional file 1 **Characterization of the gene trap allele in *Sel1l***. (**A**) Schematic representation of the wild-type (*Sel1l*^+^) and gene trap (*Sel1l*^*βgeo*^) allele in *Sel1l*. Solid black rectangles represent exons; the open box in the Sel1l^- ^allele represents the gene trap insertion. Splicing events are indicated by dashed lines. (**B**) PCR analysis of genomic DNAs from wild-type (WT), heterozygous (HT) and mutant (MU) mice using the indicated Sel1l and βgeo-specific primers (arrows). The data confirm the presence of a gene trap insertion in intron 14. (**C**) RT-PCR analysis of RNAs from E12.5 WT, HT and MU embryos using the indicated Sel1l and βgeo-specific primers (arrows). The data indicate that the gene trap allele in *Sel1l *completely blocks RNA splicing between exon 14 and 15, resulting in a truncated Sel1l transcript fused in frame with βgeo transcript. (**D**) Schematic representation of the full-length (FL) (top) and deletion mutant (DM) SEL1L peptides generated from the wild-type and gene trap Sel1l alleles. Key protein domains are shown in colored boxes. Numbers represent amino acid positions. The gene trap allele in *Sel1l *generates a fusion protein containing the N-terminal 465 amino acids of SEL1L and βgeo. The mutant peptide lacks the Hrd3-like motif, the transmembrane domain, the proline-rich domain and 4 SEL1L-like repeats. Click here for file

Additional file 2 ***Sel1l*-deficient pancreatic epithelium exhibits impaired growth, branching morphology and differentiation *ex vivo***. The dorsal pancreatic bud of wild-type (+/+) and *Sel1l*^*βgeo*/*βgeo *^(-/-) embryos were isolated at E11.5 and cultured as described in Materials and Methods. Cultured pancreatic explants were immunostained with the indicated antibodies. *Sel1l*-deficient pancreatic epithelium grows poorly and displays impaired branching morphology (**B **and **D**), as compared to wild-type control explants (**A **and **C**). Scale bar: 100 μm. Click here for file

## References

[B1] Slack JM Developmental biology of the pancreas Development 1995 121 1569 80 7600975 10.1242/dev.121.6.1569

[B2] Jorgensen MC Ahnfelt-Ronne J Hald J Madsen OD Serup P Hecksher-Sorensen J An illustrated review of early pancreas development in the mouse Endocr Rev 2007 28 685 705 10.1210/er.2007-0016 17881611

[B3] Kim SK MacDonald RJ Signaling and transcriptional control of pancreatic organogenesis Curr Opin Genet Dev 2002 12 540 7 10.1016/S0959-437X(02)00338-6 12200159

[B4] Gu G Dubauskaite J Melton DA Direct evidence for the pancreatic lineage NGN3+ cells are islet progenitors and are distinct from duct progenitors Development 2002 129 2447 57 11973276 10.1242/dev.129.10.2447

[B5] Pictet RL Clark WR Williams RH Rutter WJ An ultrastructural analysis of the developing embryonic pancreas Dev Biol 1972 29 436 67 10.1016/0012-1606(72)90083-8 4570759

[B6] Ahlgren U Jonsson J Edlund H The morphogenesis of the pancreatic mesenchyme is uncoupled from that of the pancreatic epithelium in IPF1/PDX1-deficient mice Development 1996 122 1409 16 8625829 10.1242/dev.122.5.1409

[B7] Offield MF Jetton TL Labosky PA Ray M Stein RW Magnuson MA Hogan BL Wright CV PDX-1 is required for pancreatic outgrowth and differentiation of the rostral duodenum Development 1996 122 983 95 8631275 10.1242/dev.122.3.983

[B8] Kawaguchi Y Cooper B Gannon M Ray M MacDonald RJ Wright CV The role of the transcriptional regulator Ptf1a in converting intestinal to pancreatic progenitors Nat Genet 2002 32 128 34 10.1038/ng959 12185368

[B9] Krapp A Knofler M Frutiger S Hughes GJ Hagenbuchle O Wellauer PK The p48 DNA-binding subunit of transcription factor PTF1 is a new exocrine pancreas-specific basic helix-loop-helix protein Embo J 1996 15 4317 29 8861960 PMC452157

[B10] Lynn FC Smith SB Wilson ME Yang KY Nekrep N German MS Sox9 coordinates a transcriptional network in pancreatic progenitor cells Proc Natl Acad Sci USA 2007 104 10500 5 10.1073/pnas.0704054104 17563382 PMC1965542

[B11] Seymour PA Freude KK Tran MN Mayes EE Jensen J Kist R Scherer G Sander M SOX9 is required for maintenance of the pancreatic progenitor cell pool Proc Natl Acad Sci USA 2007 104 1865 70 10.1073/pnas.0609217104 17267606 PMC1794281

[B12] Gradwohl G Dierich A LeMeur M Guillemot F neurogenin3 is required for the development of the four endocrine cell lineages of the pancreas Proc Natl Acad Sci USA 2000 97 1607 11 10.1073/pnas.97.4.1607 10677506 PMC26482

[B13] Apelqvist A Li H Sommer L Beatus P Anderson DJ Honjo T Hrabe de Angelis M Lendahl U Edlund H Notch signalling controls pancreatic cell differentiation Nature 1999 400 877 81 10.1038/23716 10476967

[B14] Naya FJ Huang HP Qiu Y Mutoh H DeMayo FJ Leiter AB Tsai MJ Diabetes defective pancreatic morphogenesis and abnormal enteroendocrine differentiation in BETA2/neuroD-deficient mice Genes Dev 1997 11 2323 34 10.1101/gad.11.18.2323 9308961 PMC316513

[B15] Naya FJ Stellrecht CM Tsai MJ Tissue-specific regulation of the insulin gene by a novel basic helix-loop-helix transcription factor Genes Dev 1995 9 1009 19 10.1101/gad.9.8.1009 7774807

[B16] Sosa-Pineda B Chowdhury K Torres M Oliver G Gruss P The Pax4 gene is essential for differentiation of insulin-producing beta cells in the mammalian pancreas Nature 1997 386 399 402 10.1038/386399a0 9121556

[B17] St-Onge L Sosa-Pineda B Chowdhury K Mansouri A Gruss P Pax6 is required for differentiation of glucagon-producing alpha-cells in mouse pancreas Nature 1997 387 406 9 10.1038/387406a0 9163426

[B18] Sussel L Kalamaras J Hartigan-O'Connor DJ Meneses JJ Pedersen RA Rubenstein JL German MS Mice lacking the homeodomain transcription factor Nkx2.2 have diabetes due to arrested differentiation of pancreatic beta cells Development 1998 125 2213 21 9584121 10.1242/dev.125.12.2213

[B19] Sander M Sussel L Conners J Scheel D Kalamaras J Dela Cruz F Schwitzgebel V Hayes-Jordan A German M Homeobox gene Nkx6.1 lies downstream of Nkx2.2 in the major pathway of beta-cell formation in the pancreas Development 2000 127 5533 40 11076772 10.1242/dev.127.24.5533

[B20] Collombat P Mansouri A Hecksher-Sorensen J Serup P Krull J Gradwohl G Gruss P Opposing actions of Arx and Pax4 in endocrine pancreas development Genes Dev 2003 17 2591 603 10.1101/gad.269003 14561778 PMC218152

[B21] Ahlgren U Pfaff SL Jessell TM Edlund T Edlund H Independent requirement for ISL1 in formation of pancreatic mesenchyme and islet cells Nature 1997 385 257 60 10.1038/385257a0 9000074

[B22] Gierl MS Karoulias N Wende H Strehle M Birchmeier C The zinc-finger factor Insm1 (IA-1) is essential for the development of pancreatic beta cells and intestinal endocrine cells Genes Dev 2006 20 2465 78 10.1101/gad.381806 16951258 PMC1560419

[B23] Johansson KA Dursun U Jordan N Gu G Beermann F Gradwohl G Grapin-Botton A Temporal control of neurogenin3 activity in pancreas progenitors reveals competence windows for the generation of different endocrine cell types Dev Cell 2007 12 457 65 10.1016/j.devcel.2007.02.010 17336910

[B24] Jensen J Pedersen EE Galante P Hald J Heller RS Ishibashi M Kageyama R Guillemot F Serup P Madsen OD Control of endodermal endocrine development by Hes-1 Nat Genet 2000 24 36 44 10.1038/71657 10615124

[B25] Fujikura J Hosoda K Iwakura H Tomita T Noguchi M Masuzaki H Tanigaki K Yabe D Honjo T Nakao K Notch/Rbp-j signaling prevents premature endocrine and ductal cell differentiation in the pancreas Cell Metab 2006 3 59 65 10.1016/j.cmet.2005.12.005 16399505

[B26] Esni F Ghosh B Biankin AV Lin JW Albert MA Yu X MacDonald RJ Civin CI Real FX Pack MA Ball DW Leach SD Notch inhibits Ptf1 function and acinar cell differentiation in developing mouse and zebrafish pancreas Development 2004 131 4213 24 10.1242/dev.01280 15280211

[B27] Hald J Hjorth JP German MS Madsen OD Serup P Jensen J Activated Notch1 prevents differentiation of pancreatic acinar cells and attenuate endocrine development Dev Biol 2003 260 426 37 10.1016/S0012-1606(03)00326-9 12921743

[B28] Murtaugh LC Stanger BZ Kwan KM Melton DA Notch signaling controls multiple steps of pancreatic differentiation Proc Natl Acad Sci USA 2003 100 14920 5 10.1073/pnas.2436557100 14657333 PMC299853

[B29] Greenwald I LIN-12/Notch signaling: lessons from worms and flies Genes Dev 1998 12 1751 62 10.1101/gad.12.12.1751 9637676

[B30] Baron M Aslam H Flasza M Fostier M Higgs JE Mazaleyrat SL Wilkin MB Multiple levels of Notch signal regulation (review) Mol Membr Biol 2002 19 27 38 10.1080/09687680110112929 11989820

[B31] Justice NJ Jan YN Variations on the Notch pathway in neural development Curr Opin Neurobiol 2002 12 64 70 10.1016/S0959-4388(02)00291-X 11861166

[B32] Cormier S Vandormael-Pournin S Babinet C Cohen-Tannoudji M Developmental expression of the Notch signaling pathway genes during mouse preimplantation development Gene Expr Patterns 2004 4 713 7 10.1016/j.modgep.2004.04.003 15465494

[B33] Haddon C Smithers L Schneider-Maunoury S Coche T Henrique D Lewis J Multiple delta genes and lateral inhibition in zebrafish primary neurogenesis Development 1998 125 359 70 9425132 10.1242/dev.125.3.359

[B34] Kaern M Elston TC Blake WJ Collins JJ Stochasticity in gene expression: from theories to phenotypes Nat Rev Genet 2005 6 451 64 10.1038/nrg1615 15883588

[B35] Fleming RJ Gu Y Hukriede NA Serrate-mediated activation of Notch is specifically blocked by the product of the gene fringe in the dorsal compartment of the Drosophila wing imaginal disc Development 1997 124 2973 81 9247339 10.1242/dev.124.15.2973

[B36] Lawrence N Klein T Brennan K Martinez Arias A Structural requirements for notch signalling with delta and serrate during the development and patterning of the wing disc of Drosophila Development 2000 127 3185 95 10862754 10.1242/dev.127.14.3185

[B37] Le Borgne R Bardin A Schweisguth F The roles of receptor and ligand endocytosis in regulating Notch signaling Development 2005 132 1751 62 10.1242/dev.01789 15790962

[B38] Lai EC Protein degradation: four E3s for the notch pathway Curr Biol 2002 12 R74 8 10.1016/S0960-9822(01)00679-0 11818085

[B39] Biunno I Castiglioni B Rogozin IB DeBellis G Malferrari G Cattaneo M Cross-species conservation of SEL1L, a human pancreas-specific expressing gene Omics 2002 6 187 98 10.1089/153623102760092788 12143964

[B40] Biunno I Appierto V Cattaneo M Leone BE Balzano G Socci C Saccone S Letizia A Della Valle G Sgaramella V Isolation of a pancreas-specific gene located on human chromosome 14q31: expression analysis in human pancreatic ductal carcinomas Genomics 1997 46 284 6 10.1006/geno.1997.5018 9417916

[B41] Donoviel DB Donoviel MS Fan E Hadjantonakis A Bernstein A Cloning and characterization of Sel-1l, a murine homolog of the C. elegans sel-1 gene Mech Dev 1998 78 203 7 10.1016/S0925-4773(98)00146-4 9858735

[B42] Su AI Cooke MP Ching KA Hakak Y Walker JR Wiltshire T Orth AP Vega RG Sapinoso LM Moqrich A Patapoutian A Hampton GM Schultz PG Hogenesch JB Large-scale analysis of the human and mouse transcriptomes Proc Natl Acad Sci USA 2002 99 4465 70 10.1073/pnas.012025199 11904358 PMC123671

[B43] Donoviel DB Bernstein A SEL-1L maps to human chromosome 14, near the insulin-dependent diabetes mellitus locus 11 Genomics 1999 56 232 3 10.1006/geno.1998.5534 10051412

[B44] Sundaram M Greenwald I Suppressors of a lin-12 hypomorph define genes that interact with both lin-12 and glp-1 in Caenorhabditis elegans Genetics 1993 135 765 83 8293978 10.1093/genetics/135.3.765 PMC1205719

[B45] Grant B Greenwald I The Caenorhabditis elegans sel-1 gene, a negative regulator of lin-12 and glp-1, encodes a predicted extracellular protein Genetics 1996 143 237 47 8722778 10.1093/genetics/143.1.237 PMC1207257

[B46] Grant B Greenwald I Structure, function, and expression of SEL-1, a negative regulator of LIN-12 and GLP-1 in C. elegans Development 1997 124 637 44 9043078 10.1242/dev.124.3.637

[B47] Rooman I De Medts N Baeyens L Lardon J De Breuck S Heimberg H Bouwens L Expression of the Notch signaling pathway and effect on exocrine cell proliferation in adult rat pancreas Am J Pathol 2006 169 1206 14 10.2353/ajpath.2006.050926 17003479 PMC1698841

[B48] Hosokawa N Wada I Nagasawa K Moriyama T Okawa K Nagata K Human XTP3-B forms an endoplasmic reticulum quality control scaffold with the HRD1-SEL1L ubiquitin ligase complex and BiP J Biol Chem 2008 283 20914 24 10.1074/jbc.M709336200 18502753 PMC3258950

[B49] Mueller B Lilley BN Ploegh HL SEL1L, the homologue of yeast Hrd3p, is involved in protein dislocation from the mammalian ER J Cell Biol 2006 175 261 70 10.1083/jcb.200605196 17043138 PMC2064567

[B50] Zecchin E Filippi A Biemar F Tiso N Pauls S Ellertsdottir E Gnugge L Bortolussi M Driever W Argenton F Distinct delta and jagged genes control sequential segregation of pancreatic cell types from precursor pools in zebrafish Dev Biol 2007 301 192 204 10.1016/j.ydbio.2006.09.041 17059815

[B51] Wessells NK Evans J Ultrastructural studies of early morphogenesis and cytodifferentiation in the embryonic mammalian pancreas Dev Biol 1968 17 413 46 10.1016/0012-1606(68)90073-0 5650009

[B52] Rall LB Pictet RL Williams RH Rutter WJ Early differentiation of glucagon-producing cells in embryonic pancreas: a possible developmental role for glucagon Proc Natl Acad Sci USA 1973 70 3478 82 10.1073/pnas.70.12.3478 4519640 PMC427263

[B53] Sundaram M Greenwald I Genetic and phenotypic studies of hypomorphic lin-12 mutants in Caenorhabditis elegans Genetics 1993 135 755 63 8293977 10.1093/genetics/135.3.755 PMC1205718

[B54] Chiaramonte R Calzavara E Basile A Comi P Sherbet GV Notch signal transduction is not regulated by SEL1L in leukaemia and lymphoma cells in culture Anticancer Res 2002 22 4211 4 12553058

[B55] Mukherjee A Veraksa A Bauer A Rosse C Camonis J Artavanis-Tsakonas S Regulation of Notch signalling by non-visual beta-arrestin Nat Cell Biol 2005 7 1191 201 10.1038/ncb1327 16284625

[B56] Chastagner P Israel A Brou C AIP4/Itch regulates Notch receptor degradation in the absence of ligand PLoS ONE 2008 3 e2735 10.1371/journal.pone.0002735 18628966 PMC2444042

[B57] Cattaneo M Otsu M Fagioli C Martino S Lotti LV Sitia R Biunno I SEL1L and HRD1 are involved in the degradation of unassembled secretory Ig-mu chains J Cell Physiol 2008 215 794 802 10.1002/jcp.21364 18314878

[B58] Cormier JH Tamura T Sunryd JC Hebert DN EDEM1 recognition and delivery of misfolded proteins to the SEL1L-containing ERAD complex Mol Cell 2009 34 627 33 10.1016/j.molcel.2009.05.018 19524542 PMC2740909

[B59] Mueller B Klemm EJ Spooner E Claessen JH Ploegh HL SEL1L nucleates a protein complex required for dislocation of misfolded glycoproteins Proc Natl Acad Sci USA 2008 105 12325 30 10.1073/pnas.0805371105 18711132 PMC2527910

[B60] Oresic K Mueller B Tortorella D Cln6 mutants associated with neuronal ceroid lipofuscinosis are degraded in a proteasome-dependent manner Biosci Rep 2009 29 173 81 10.1042/BSR20080143 18811591 PMC2674128

[B61] Burlison JS Long Q Fujitani Y Wright CV Magnuson MA Pdx-1 and Ptf1a concurrently determine fate specification of pancreatic multipotent progenitor cells Dev Biol 2008 316 74 86 10.1016/j.ydbio.2008.01.011 18294628 PMC2425677

[B62] Matsuoka TA Zhao L Artner I Jarrett HW Friedman D Means A Stein R Members of the large Maf transcription family regulate insulin gene transcription in islet beta cells Mol Cell Biol 2003 23 6049 62 10.1128/MCB.23.17.6049-6062.2003 12917329 PMC180917

[B63] Percival AC Slack JM Analysis of pancreatic development using a cell lineage label Exp Cell Res 1999 247 123 32 10.1006/excr.1998.4322 10047454

[B64] Cheng HT Miner JH Lin M Tansey MG Roth K Kopan R Gamma-secretase activity is dispensable for mesenchyme-to-epithelium transition but required for podocyte and proximal tubule formation in developing mouse kidney Development 2003 130 5031 42 10.1242/dev.00697 12952904

