# Supplementary figures and images for: SEL1L deficiency impairs growth and differentiation of pancreatic epithelial cells
Source: BMC Dev Biol. 2010 Feb 19;10:19. doi: 10.1186/1471-213X-10-19 (PMC2848149; doi:10.1186/1471-213X-10-19)

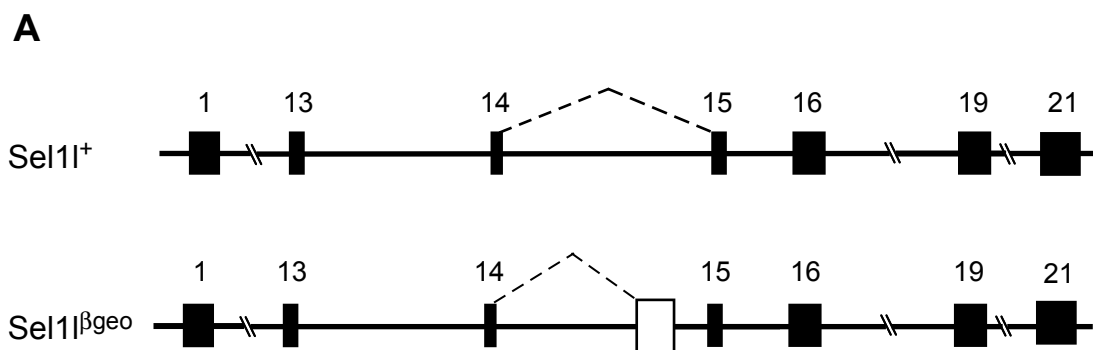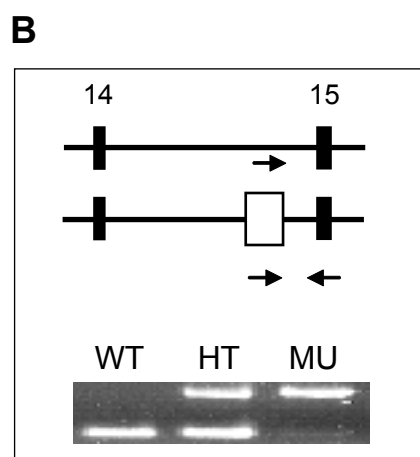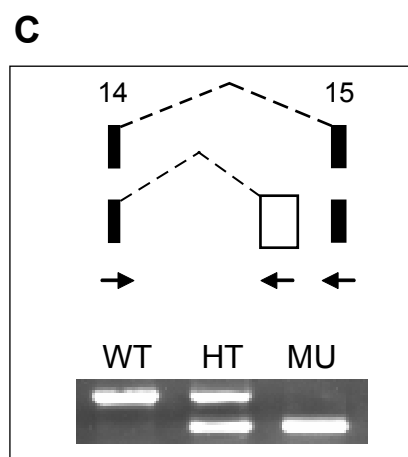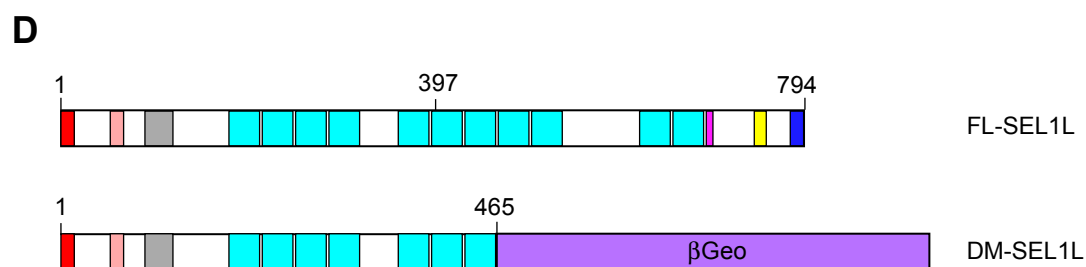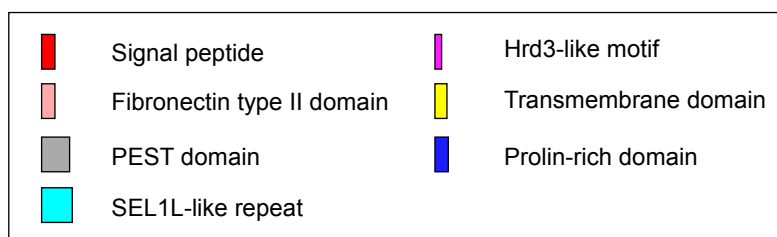

Supplement: Additional file 1 — Characterization of the gene trap allele in Sel1l. (A) Schematic representation of the wild-type (Sel1l+) and gene trap (Sel1lβgeo) allele in Sel1l. Solid black rectangles represent exons; the open box in the Sel1l- allele represents the gene trap insertion. Splicing events are indicated by dashed lines. (B) PCR analysis of genomic DNAs from wild-type (WT), heterozygous (HT) and mutant (MU) mice using the indicated Sel1l and βgeo-specific primers (arrows). The data confirm the presence of a gene trap insertion in intron 14. (C) RT-PCR analysis of RNAs from E12.5 WT, HT and MU embryos using the indicated Sel1l and βgeo-specific primers (arrows). The data indicate that the gene trap allele in Sel1l completely blocks RNA splicing between exon 14 and 15, resulting in a truncated Sel1l transcript fused in frame with βgeo transcript. (D) Schematic representation of the full-length (FL) (top) and deletion mutant (DM) SEL1L peptides generated from the wild-type and gene trap Sel1l alleles. Key protein domains are shown in colored boxes. Numbers represent amino acid positions. The gene trap allele in Sel1l generates a fusion protein containing the N-terminal 465 amino acids of SEL1L and βgeo. The mutant peptide lacks the Hrd3-like motif, the transmembrane domain, the proline-rich domain and 4 SEL1L-like repeats. [file 1471-213X-10-19-S1.PDF]

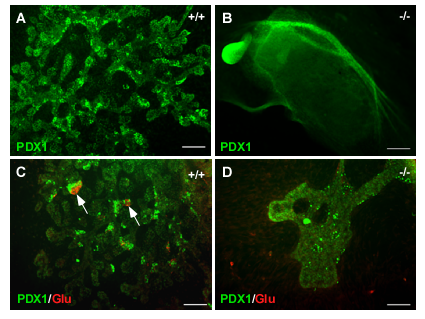

Supplement: Additional file 2 — Sel1l-deficient pancreatic epithelium exhibits impaired growth, branching morphology and differentiation ex vivo. The dorsal pancreatic bud of wild-type (+/+) and Sel1lβgeo/βgeo (-/-) embryos were isolated at E11.5 and cultured as described in Materials and Methods. Cultured pancreatic explants were immunostained with the indicated antibodies. Sel1l-deficient pancreatic epithelium grows poorly and displays impaired branching morphology (B and D), as compared to wild-type control explants (A and C). Scale bar: 100 μm. [file 1471-213X-10-19-S2.TIFF]
